# Live and Diet by Your Gut Microbiota

**DOI:** 10.1128/mBio.02335-19

**Published:** 2019-10-08

**Authors:** Christine M. Bassis

**Affiliations:** a Division of Infectious Diseases, Department of Internal Medicine, University of Michigan, Ann Arbor, Michigan, USA

**Keywords:** diet, gut microbiota, infection, sepsis

## Abstract

Diet influences health in multiple ways. One important effect of diet is on the gut microbiota. The effects of diet are often related to an individual’s specific microbiota composition. The close links between health, diet, and gut microbiota are illustrated in a new mouse model of sepsis where the combination of a high-fat/low-fiber Western diet, antibiotics, and surgery promotes the development of lethal sepsis. Diet can also influence infection via the gut microbiota beyond sepsis. Future studies with this model may inform the use of microbiota analysis and personalized diets to protect surgery patients from infection and sepsis.

## Abstract (precis)

Diet influences health in multiple ways. One important effect of diet is on the gut microbiota. The effects of diet are often related to an individual’s specific microbiota composition. The close links between health, diet, and gut microbiota are illustrated in a new mouse model of sepsis where the combination of a high-fat/low-fiber Western diet, antibiotics, and surgery promotes the development of lethal sepsis. Diet can also influence infection via the gut microbiota beyond sepsis.

## COMMENTARY

It is no surprise that our diet affects our health and our gut microbiota (1). A high-fat/low-fiber Western diet can contribute to numerous conditions, including obesity, type 2 diabetes, and cardiovascular disease. However, the relationship between health and diet is complex, beginning with the question of what constitutes a “healthy” diet.

**FIG 1:**
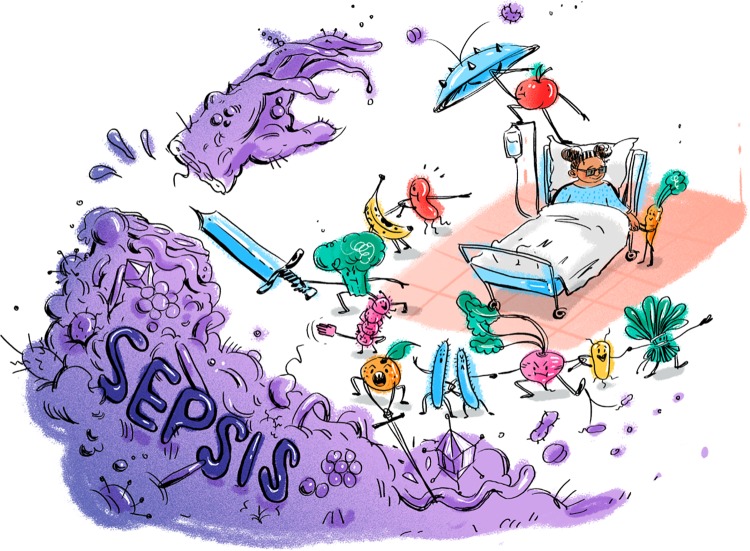
A healthy diet and gut microbiota team up to protect patients against sepsis. Continued work in models like the one developed by Hyoju et al. and in human cohorts may lead to a future where personalized diets that work with an individual’s microbiota are used as tools to prevent sepsis and other infections. (Illustration by Patrick Lane, Sceyence Studios.)

In the health care setting, a healthy diet may be defined as one that improves specific patient outcomes. Hospitals already provide a variety of customized diets, but what if a patient’s diet could also be optimized to help prevent infections or sepsis (Fig. 1)?

Part of the complexity in the relationship between health and diet stems from the variation between individuals in response to food. Recent studies of human cohorts have demonstrated high variation in postmeal glucose levels between individuals eating the same meals (2, 3). Interestingly, gut microbiota composition is one key factor that associates with an individual’s postmeal glucose response.

The gut microbiota is an important layer in the relationship between diet and health. The effects of dietary changes also vary by individual and, in many cases, associate with specific gut microbiota members or compositions. For example, in healthy college students, supplementation with resistant starch yielded varied responses in terms of fecal butyrate levels and microbiota alteration related to an individual’s baseline (4). Mouse models also demonstrate the relationship between the outcome of diet change and the microbiota. In one study, transferring mice from a high-fat diet to standard chow resulted in the convergence of several metabolic phenotypes (including body fat content, glucose tolerance, and serum insulin levels) with those of control mice that were always fed standard chow (11). However, the microbiota of the mice initially fed a high-fat diet remained distinct and was associated with faster weight gain upon returning to a high-fat diet. In another study, feeding laboratory mice a diet low in microbiota-accessible carbohydrates (MACs) over multiple generations caused the apparent extinction of some members of the gut microbiota (5). After multiple generations on the low-MAC diet, switching mice to a diet high in MACs did not restore their ancestral microbiota.

A new article by Hyoju et al. highlights the interplay between diet, gut microbiota, and health with a mouse model of lethal sepsis that requires a Western (high-fat/low-fiber) diet, antibiotic treatment, and surgery (6).

Sepsis, infection-induced organ dysfunction, affects approximately 1.7 million people in the United States each year and results in an estimated 270,000 deaths (https://www.cdc.gov/sepsis/datareports/index.html). However, sepsis is poorly understood, and by the time it is recognized, available treatments are too often ineffective. Improved prevention and treatment strategies are needed. In the mouse model developed by Hyoju et al., 3 components are required for the development of lethal sepsis: Western diet, antibiotic treatment, and surgery (30% hepatectomy). The requirement for all 3 components suggests possible opportunities for intervention and prevention.

In this model, the majority of mice on the high-fat/low-fiber Western diet that received antibiotics (cefoxitin and clindamycin) followed by surgery (30% hepatectomy) developed signs of sepsis and did not survive. In contrast, most mice on standard chow that received antibiotics prior to surgery did not develop signs of sepsis and survived. All mice that did not receive antibiotics prior to surgery survived, regardless of diet. The pattern of disease severity between groups was associated with levels of bacteria in the blood, liver, and spleen. Some specific members of the gut microbiota that ended up in the blood, liver, and spleen were enriched in the gut (cecal tissue) with Western diet and antibiotics.

Both antibiotics and diet alter the microbiota. In the model developed by Hyoju et al., the combination of Western diet and antibiotics dramatically increased the relative abundances of *Proteobacteria* in both the gut lumen and cecal tissue. Serratia marcescens, one of the organisms that ended up in the blood, liver, and spleen, was especially enriched in the cecal tissues of mice receiving a Western diet and antibiotics. The Western diet was not sufficient for postsurgery sepsis development without antibiotics, likely because the sepsis-inducing organisms were enriched by the combination of antibiotics and Western diet. Another consequence of the Western diet in the model developed by Hyoju et al. was an increase in the antibiotic resistance of the microbiota. This aspect of the model was intriguing because it appeared to be independent of the antibiotics used in the model but may represent problems in attempts to treat sepsis.

In addition to enriching gut bacteria with the potential to induce sepsis, the Western diet may promote postsurgery sepsis via effects directly on the host. In a second recently developed model of sepsis, mice fed a high-fat/low-fiber diet developed more-severe sepsis with lipopolysaccharide (LPS) administration than mice fed standard chow even in the absence of a microbiota (7). In this model, the high-fat/low-fiber diet altered the immune system and increased gut permeability.

There are also links between diet, microbiota, and infection outside sepsis. In another mouse model, gnotobiotic mice colonized with a defined microbiota isolated from the human gut were fed fiber-rich or fiber-free diets (8). The fiber-free diet altered the community composition compared to that of the fiber-rich diet, increasing the relative abundances of known mucus degraders, which increased the numbers of transcripts for enzymes involved in mucus degradation, resulting in degradation of the mucus layer and increased susceptibility to infection with Citrobacter rodentium. Switching between the fiber-rich and fiber-free diets from day to day caused large shifts in the relative abundances of some members of the community, including large increases in the relative abundances of known mucus degraders with the fiber-free diet. Hyperglycemia, high blood glucose levels, may also contribute to increased microbial dissemination with a Western diet. In a mouse model, hyperglycemia increased gut permeability and dissemination from the gut of a pathogen and probably of other members of the microbiota (9). In humans, levels of microbial products in the blood correlated with long-term glucose levels measured by hemoglobin A1c (9).

Diet changes can measurably alter the composition of the human gut microbiota within days. In one study, the gut microbiotas of humans were significantly altered only 2 days after the subjects switched to an animal-derived diet (10). When the subjects returned to their regular diets after 4 days of the animal-derived diet, their gut microbiotas returned to a composition similar to the baseline composition.

Although short-term diet changes can alter the microbiota, it is unknown whether short-term diet changes have health benefits. Future studies using the model developed by Hyoju et al. might investigate whether diet changes are able to protect against the development of sepsis, either independently or in concert with microbiota changes. For example, could mice that have been on a high-fat/low fiber Western diet be protected by changing to standard chow before surgery? If so, how long would mice need to be on standard chow before surgery? What specific components of standard chow are protective? Additionally, different antibiotics could be tested to determine if the potential benefits of a diet change depend on the specific microbiota of the mice.

If specific diets can protect against sepsis and the protections translate to humans, microbiota-guided dietary interventions could become a standard part of patient care prior to surgeries, with potential benefits for sepsis as well as other infections where gut colonization precedes infection.
